# Molecular Biomarkers in Cancer

**DOI:** 10.3390/biom12081021

**Published:** 2022-07-23

**Authors:** Virinder Kaur Sarhadi, Gemma Armengol

**Affiliations:** 1 Department of Oral and Maxillofacial Diseases, Helsinki University Hospital and University of Helsinki, 00290 Helsinki, Finland; virinder.sarhadi@helsinki.fi; 2 Department of Animal Biology, Plant Biology, and Ecology, Faculty of Biosciences, Universitat Autònoma de Barcelona, 08193 Barcelona, Catalonia, Spain

**Keywords:** cancer biomarkers, biomolecules, risk assessment, diagnostic biomarkers, predictive biomarkers

## Abstract

Molecular cancer biomarkers are any measurable molecular indicator of risk of cancer, occurrence of cancer, or patient outcome. They may include germline or somatic genetic variants, epigenetic signatures, transcriptional changes, and proteomic signatures. These indicators are based on biomolecules, such as nucleic acids and proteins, that can be detected in samples obtained from tissues through tumor biopsy or, more easily and non-invasively, from blood (or serum or plasma), saliva, buccal swabs, stool, urine, etc. Detection technologies have advanced tremendously over the last decades, including techniques such as next-generation sequencing, nanotechnology, or methods to study circulating tumor DNA/RNA or exosomes. Clinical applications of biomarkers are extensive. They can be used as tools for cancer risk assessment, screening and early detection of cancer, accurate diagnosis, patient prognosis, prediction of response to therapy, and cancer surveillance and monitoring response. Therefore, they can help to optimize making decisions in clinical practice. Moreover, precision oncology is needed for newly developed targeted therapies, as they are functional only in patients with specific cancer genetic mutations, and biomarkers are the tools used for the identification of these subsets of patients. Improvement in the field of cancer biomarkers is, however, needed to overcome the scientific challenge of developing new biomarkers with greater sensitivity, specificity, and positive predictive value.

## 1. Introduction

A cancer biomarker is a characteristic that is measured as an indicator of risk of cancer, occurrence of cancer, or patient outcome. These characteristics can be either molecular, cellular, physiologic, or imaging-based. The present review focuses on molecular (and cellular) cancer biomarkers. These biomolecules, found in tissues or body fluids, are present or produced by cancer cells or normal cells in response to cancer. On the one hand, biomarker testing in cancer involves profiling tumor or body fluids to detect changes in DNA, RNA, proteins, or other biomolecules that provide information for cancer diagnosis, prognosis, precision medicine/guiding cancer treatment, predicting drug response, or cancer monitoring. On the other hand, genetic testing, which is different from cancer biomarker testing, is used for detecting germline genetic variations associated with cancer susceptibility, hereditary cancer, or cancer-associated syndromes [1]. Germline genetic markers can, in addition to providing cancer susceptibility information, also provide useful information regarding treatment options [2]. They can also be included as cancer biomarkers in a broader sense. In the following sections, we discuss the numerous molecular changes that are useful as cancer biomarkers, explaining the different types of biomolecules, types of samples, and the techniques used for detecting cancer biomarkers. We also discuss different applications of cancer biomarkers in clinics and the steps involved in the process from cancer biomarker discovery to their clinical implementation.

## 2. Cancer-Associated Alterations

### 2.1. Germline Genetic Variants

There are certain inherited or germline variants that render individuals carrying them a higher risk of developing cancer. Germline variants can be classified into three groups according to their frequency and their effect size to cause disease: rare variants with high penetrance, moderately frequent variants with moderate penetrance, and common variants with low penetrance. The first ones correspond to cancer-predisposing syndromes and hereditary cancers and are good candidates to be used as cancer risk assessment biomarkers, because of their strong effect. For example, it is well-known that *BRCA1* and *BRCA2* high-penetrance variants are strongly linked to breast and ovarian cancer. Germline variants can have different penetrance for different cancer types; for example, Lynch syndrome associated variants in genes *EPCAM*, *MLH1*, *MLH2*, *MSH6*, and *PMS2* have higher penetrance for colorectal cancer (CRC) than for pancreatic cancer [3]. Moreover, the risk of cancer can be different for different genes [3]. Germline genetic markers are not only useful for identifying cancer susceptibility but are also important prognostic and predictive markers for targeted therapies. For example, poly (ADP-ribose) polymerase inhibitors are effective for germline *BRCA* mutant breast and ovarian cancer [4].

A large cancer study on 10,389 cases and 33 cancer types reported pathogenic germline variants frequency of 8% among cancer patients and discovered 853 pathogenic variants [5]. Next-generation sequencing (NGS) of the tumor sample can be used to detect germline variants, besides somatic mutations. A recent study on more than 21,000 cancer patients using a Food and Drug Administration (FDA)-approved NGS panel and pipeline, showed that tumor-only sequencing identified 89.5% of pathogenic germline variants, while missing mainly germline copy number variations, intronic variants, and repetitive element insertions [6]. Recent studies highlight the relevance of studying germline markers, along with somatic tumor markers, as germline pathological variants have been found in patients with no family history of cancer [3]. Moreover, it increases the germline variant detection in familial cancer patients, which would otherwise not have been identified [7]. However, it also increases the frequency of variants of unknown significance, which can make the interpretation of results difficult.

### 2.2. Somatic Genetic Mutations

Genomic instability is an important feature of cancer cells, driving cancer evolution and its adaptation to changing microenvironment. Most cancers result from the accumulation of somatic mutations, some of which are specific to a cancer type, while others are shared with other cancers. The alterations can involve a wide range of sizes, from a large part or a whole chromosome to single base pair changes. Chromosomal abnormalities comprise numerical abnormalities (aneuploidies and polyploidies) and structural abnormalities (translocations; inversions; and copy number alterations (CNAs), including insertions and deletions, as well as chromothripsis, which can result in massive rearrangements [8]). Those chromosomal abnormalities that are specific and recurrent are relevant cancer biomarkers, and they are used mostly in hematologic malignancies. For example, the Philadelphia chromosome was the first chromosomal abnormality to be detected in cancer, involving translocation of chromosomes 22 and 9, resulting in *BCR*–*ABL* fusion. It is commonly seen in chronic myeloid leukemia, but can occur in acute myeloid leukemia, too, and is used for their diagnosis. A comprehensive list of gene fusions obtained from an analysis of RNA sequencing and DNA copy number data from The Cancer Genome Atlas is available at https://tumorfusions.org/ (accessed on 22 February 2022).

However, the more common genetic alterations used as cancer biomarkers are mutations involving a single nucleotide (single nucleotide variants or SNVs) or a few nucleotides (small insertions and deletions or indels), e.g., driver mutations in *EGFR*, *KRAS*, *BRAF*, *TP53*, *KITK*, and other genes. Results from the Pan-Cancer Gene Atlas sequencing project [9] on 3281 tumors from 12 cancer types identified 127 recurrently mutated genes. The mutation frequency was found to depend on tumor types ranging from 0.28 mutations/Mb in acute myeloid leukemia to 8.15 in lung squamous cell carcinoma. Despite the large number of mutations seen in each tumor, only four or five mutations are thought to be drivers of cancer development [10]. A more recent and detailed analysis of Pan-Cancer data from 33 different cancer types identified 299 driver genes and around 3400 driver mutations based on in silico methods together with experimental validation [11]. Mutations in *TP53* were found to be the most commonly shared among 27 different cancer types, followed by *PIK3CA*, *KRAS*, *PTEN*, and *ARID1A* (in 15 or more). However, the majority of the genes (142) were found mutated in only one cancer type. Interestingly, they reported that 57% of tumors had mutations that could be targeted by known cancer treatments [11]. A detailed description of somatic mutations in tumor tissue in different cancers can be found in a manually curated COSMIC (Catalogue of Somatic Mutations in Cancer) database (https://cancer.sanger.ac.uk/cosmic, accessed on 22 February 2022), which also includes separate datasets for gene mutations with causal implications in cancer, cancer-driving gene mutations, and mutations actionable with precision oncology.

In addition to mutations seen in tumor tissue, cancer patients have cell-free DNA originating from cancer cell lysis/death or active secretion, which is referred to as circulating tumor DNA (ctDNA). It can be found in body fluids such as blood, urine, stool, saliva, sputum, and exhaled breath, and reflects genomic alterations similar to those seen in tumor DNA [12,13,14]. Moreover, healthy and cancer patients can show differences in ctDNA concentration, fragment size, and the relative ratio of mitochondrial/nuclear DNA, making ctDNA a good potential cancer biomarker. The main challenge associated with the detection of ctDNA in plasma or other body fluids is its very low concentration, thus requiring very sensitive methods for its detection.

Overall, it is important to consider the factors that can affect DNA mutation detection, which include tumor DNA content in total DNA, sample type (e.g., fresh frozen or formalin-fixed paraffin-embedded (FFPE) tumor tissue, or body fluids, which can harbor inhibitors or factors affecting detection efficiency), as well as the detection technique used. Most of the small DNA alterations are readily studied by DNA sequencing or polymerase chain reaction (PCR)-based methods, while those involving larger DNA fragments are studied by methods such as fluorescent in situ hybridization (FISH), array comparative genomic hybridization, or similar methods. In the case of gene fusions, the RNA transcribed from the gene fusion can be used for PCR- or NGS-based diagnosis. RNA fusion panels are now available to test the most common fusions in tumors by using NGS, while single gene fusions can be tested by reverse transcription PCR [15]. Finally, it is important to consider that tumors can be highly heterogeneous; therefore, developing good cancer biomarkers requires a multiple gene approach. Furthermore, cancer patients have their specific tumor mutation profiles, and individualized approaches to treatment based on tumor profiling are being increasingly carried out (see sections below).

### 2.3. Epigenetic Variants

Epigenetic variants cause changes in DNA methylation or histone protein modifications, without affecting the coding sequence of DNA. However, they affect the structure and stability of DNA and play an important role in carcinogenesis. These epigenetic changes in cancer cells are therefore useful as cancer biomarkers, especially since DNA methylation changes occur in the early stages of tumorigenesis [16]. Loss of global DNA methylation is common in many tumors and is associated with genomic instability, DNA damage, and reactivation of transposons and retroviruses. Moreover, more localized changes in DNA methylation at the CpG-rich promoter regions of the genes can inactivate tumor suppressor genes. For example, the CpG island methylator phenotype, characterized by hypermethylation of multiple sites, is a feature associated with genomic instability and cancer [16]. Methylation of *CACN3A1G*, *IGF2*, *NEUROG1*, *RUNX3*, and *SOCS1* promoters is common in this phenotype and is associated with *MLH1* methylation and microsatellite instability. It is noteworthy that *MLH1* methylation is a biomarker used for cancer-prone Lynch syndrome testing in clinics [16]. In addition, DNA methylation biomarkers are also useful for predicting cancer treatment response; for example, tumor *MGMT* (gene important in DNA repair) promotor hypermethylation is associated with good response to alkylating drugs and used in clinical testing in glioblastomas [17].

Interestingly, these epigenetic variants can also be detected in minimally invasive samples such as plasma, where the DNA-methylation-based screening biomarkers have advantages compared to mutation detection [18]. These include their higher sensitivity and specificity in detecting early stages of cancer and in detecting residual disease, as these changes occur early on and are tissue specific. A recently developed plasma DNA methylation panel “PanSeer” comprising 477 differentially methylated regions (10,613 CpG sites) in overall cancer showed high sensitivity (88%) and specificity (96%) in detecting five common cancer types, up to four years before conventional diagnosis, in a Taizhou Longitudinal Study on 123,115 individuals who had donated plasma for long-term storage and study [19]. FDA-approved methylation-based biomarkers include *SEPT9* from plasma (Epi ProColon) and a combination of *NDRG4* and *BMP3* from stool samples in CRC.

While most methods dedicated to identifying epigenetic variants rely on bisulfite conversion of unmethylated cytosines into uracils, such as methylation-specific PCR, methylation-sensitive high-resolution melting, pyrosequencing, methylation-specific droplet digital PCR, microarray, and NGS, other non-bisulfite treatment methods are also in use, such as methylated DNA immunoprecipitation or the use of methylation-sensitive restriction enzymes.

Even though genome-wide methylome studies have identified numerous differentially methylated genes in cancer patients, it is worth mentioning that these studies have been usually carried out on small sample sizes and that they may require a standardization of methods and proper bioinformatics analysis, especially when sequencing techniques are used [20]. Validation in larger sets of patients and the development of new assays can bring many more assays to be used in clinical settings.

### 2.4. Transcriptional Alterations

The human transcriptome includes both coding or messenger RNA (mRNA) and non-coding RNA (ncRNA). Among the ncRNA, long ncRNA (lncRNA), with transcript size greater than 200 nucleotides, forms the largest group and includes long intergenic RNA, antisense RNA, pseudogenes, and circular RNAs (circRNA), while small ncRNAs include microRNA (miRNA), small interfering RNA, small nucleolar RNA, ribosomal RNA, transfer RNA, and piwi-interacting RNA. Both coding and ncRNA have been found to be differentially expressed in cancer and play a significant role in carcinogenesis. Moreover, some of these cancer-related RNA molecules can be found cell-free, and then they are called ctRNA.

#### 2.4.1. mRNAs

Tumor mRNA profiling has shown differential expression of genes in tumors compared to normal tissue and also between various histological subtypes, stages of cancer, and other tumor features. Tumors can be classified into molecular subtypes based on the RNA profile, and these molecular subtypes, irrespective of tumor type, can predict treatment response. Tumor immune profile or expression of immune-related genes are also important biomarkers for immunotherapy response. Moreover, expressions of tissue- or tumor-specific genes, mutated genes, amplified genes, or gene fusions are all useful RNA-based cancer biomarkers. In a recent transcriptome-wide analysis of plasma samples of cancer and non-cancer patients, 23 tissue- and cancer-specific ctRNA biomarkers were identified after filtering out transcripts expressed in non-cancer patients [21]. The study found that RNA expression in plasma correlated with that in matched tissue and could predict the origin of tumor tissue and cancer type.

#### 2.4.2. miRNAs

MiRNAs are small ncRNAs, around 22 nucleotides long, which regulate post-transcriptional gene expression. It is reported that each tissue expresses around 1000 miRNAs, 143 of which are found in all tissues [22]. There is enormous amount of data available related to differentially expressed miRNAs in tumor tissues (reviewed in Reference [23]), as well as in the body fluids of cancer patients, compared to normal tissue or healthy individuals, indicating their usefulness in diagnosis/differential diagnosis, prognosis, or as predictive cancer biomarkers. Moreover, miRNAs can be oncogenic, as, for example, *miR-21* and *miR-155* are overexpressed in many cancers; or they can be tumor suppressive, e.g., *let-7*, *miR-128b*, *miR-15*, and *miR-16*, which are under-expressed as a result of deletion, methylation, or other mechanisms (reviewed in Reference [23]). Moreover, miRNAs also play a role in metastasis; for example, *miR-10b* and *miR-655* can affect the tumor microenvironment by modulating immune cells or angiogenesis [24].

However, miRNA analysis can be affected by factors such as sample collection/RNA stabilization, RNA isolation method (miRNA or total RNA), analysis method (reverse transcription PCR, microarray, and sequencing), and selection of reference gene [25], all of which can affect their expression profile.

The main advantage of miRNAs as cancer biomarkers is their small size, which makes them suitable for samples with low RNA quality, such as archival FFPE samples or body fluids. Interestingly, miRNAs with differential expression in tumor tissue can also be detected in ctRNA in body fluids. Moreover, miRNAs constitute the main cargo of extracellular vesicles (EVs), where they are protected from RNases and are thus increasingly being studied for their utility as cancer biomarkers in blood (Table 1). For example, *miR-122* is found to be highly expressed in tumor tissue and serum EVs of CRC patients, especially with liver metastasis [26]. The miRNA expression profile of serum/plasma-EVs can be different from that of plasma/serum-ctRNA, and the EV-miRNA profile is found to be more informative as a cancer biomarker [27]; however, some studies suggest studying both [28]. Overall, EV-associated miRNAs are promising liquid-biopsy-based cancer biomarkers [29].

#### 2.4.3. CircRNAs

CircRNAs are endogenous lncRNAs acting as miRNA sponges and regulating transcription and splicing. They are single-stranded RNAs lacking cap and poly-A tail, with ends joined covalently to form a circular structure. Their high stability, tissue-specific expression, and association with tumor progression make them suitable candidates for cancer biomarkers. Recent studies have shown their possibility as diagnostic biomarkers, especially from EVs in body fluids (reviewed in Reference [77]).

#### 2.4.4. Other lncRNAs

Tissue specificity and association of certain lncRNAs’ expression with stage, metastasis, and survival make them candidate cancer biomarkers. Some of the lncRNA with good potential in cancer diagnosis or prognosis include *PCA3*, *MALAT1*, *HOTAIR*, *H19*, and *CCAT1* (reviewed in Reference [78]). For example, high expression of *HOTAIR*, *MALAT1*, and *CCAT2* is related to poor prognosis in various cancer types. *PCA3* is a clinically approved biomarker with high sensitivity and specificity for the detection of early prostate cancer from urine samples, while the *MALAT1* assay is more useful in patients with borderline prostate-specific antigen (PSA) levels. Plasma levels of *H19* in breast cancer, and *H19*, *HOTAIR*, and *MALAT1* in gastric cancer have shown high diagnostic potential, while single nucleotide polymorphisms in the *H19* gene have applications in cancer risk prediction. Some current clinical trials are looking for their future potential application in cancer diagnosis.

#### 2.4.5. Summary of Transcriptional Alterations

According to a manually curated database “CRMarker”, created for cancer RNA biomarker discovery [79], the top RNA diagnostic/prognostic candidate biomarkers identified from different cancer studies include the following mRNAs: *TP53*, *EGFR*, *ERBB2*, *WT1*, *CDKN2A*, *MK167*, *TERT*, *PCA3*, *PTEN*, *CD44*, *BCL2*, *ERCC1*, *CCND1*, *MET*, and *BIRC5*. Among the ncRNA, the following miRNAs are included: *miR-21*, *miR-155*, *miR-221*, *miR-210*, *miR-145*, *miR-375*, *miR-205*, *miR-126*, *miR-223*, *miR-200c*, *miR-141*, and *miR-31.* The lncRNAs are *MALAT1*, *HOTAIR*, *UCA1*, *PVT1*, *H19*, *NEAT1*, *GASS*, *lnc-FOXB2*, *lnc-BMP6-106*, *lnc-FGF1-9*, *CYTOR*, *TUG1*, and *CDKN2B-AS1;* and the circRNAs are *circ_002059*, *circ-PVT1*, *ciRS-7*, *circ_0001649*, *circ_0005075*, and *circ_100338*. In addition, levels of *BIRC5* mRNA are also increased in the serum of CRC patients, and very high levels of this mRNA are correlated with poor prognosis [80].

### 2.5. Proteomic Changes

**Figure 1:**
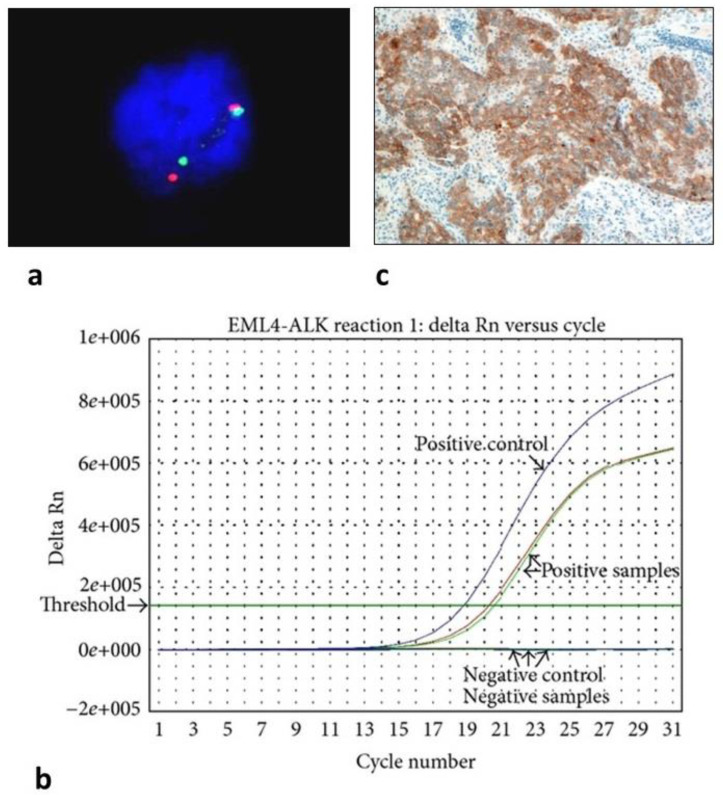
Different biomolecules for detecting cancer biomarkers. *ALK* fusions in lung tumor tissue detected as (**a**) DNA by fluorescence in situ hybridization, (**b**) RNA by reverse-transcription polymerase chain reaction, and (**c**) protein by immunohistochemistry. Figure modified from Tuonen et al. [15], (open access) under Creative Commons Attribution License.

Cancer-associated alterations at DNA and RNA levels are also observed at the protein level (Figure 1), although gene expression does not necessarily correlate with protein expression. Proteins are more difficult to study than nucleic acids, as they are complex and sensitive to physiological changes, and their function is dependent on post-translational modifications. Protein biomarkers in cancer include overexpressed proteins (e.g., HER2), mutated proteins (including neoantigens and products of gene fusions), or proteins with tumor-specific post-translational modifications (e.g., glycoproteins), all of which can be detected in tumor tissue. On the other hand, protein biomarkers detectable in blood or other body fluids, in addition to those detected in tumors, also include tissue/cell-specific proteins that have increased levels in body fluids compared to normal, e.g., PSA in the plasma of prostate cancer patients. However, the challenge for protein biomarkers from plasma/blood is the dominant expression of certain normal proteins that mask the very low expression of cancer-related proteins or protein modifications, making their characterization difficult.

Protein biomarkers were among the first to be used in cancer diagnostics. Most of them are based on cancer antigens, enzymes, and hormones, but also on changes in protein glycosylation profile, which is a characteristic feature of cancer. Glycans are polymers of monosaccharides that can conjugate with proteins to form glycoproteins. These differentially expressed glycans or glycoproteins are useful cancer biomarkers in tumor tissue, as well as in blood. The alterations in protein glycosylation can be due to the altered expression of glycoproteins or changes in the glycans or glycotransferases. Some of the glycans/glycoproteins used as cancer biomarkers include AFP, β-hCG, cancer antigen (CA)15-3, CA19-9, CA27.29, CA125, CA549, Carcinoembryonic Antigen (CEA), CEACAMS HER2, onfFN, PLAP, PSA, sTn antigen, TAG-72, TG, and Tn antigen (reviewed in Reference [81]).

Two initiatives have been launched to gain insight into proteome characterization in cancer. First, Clinical Proteomic Tumor Analysis Consortium (https://proteomics.cancer.gov, accessed on 22 February 2022) was set up to increase the understanding of cancer proteomics in relation to cancer genomics, as well as to standardize proteomic assays for clinical use. Second, the Human Protein Atlas (https://www.proteinatlas.org/humanproteome/tissue/cancer, accessed on 28 February 2022) documents all human proteins expressed in different healthy cells and tissues, as well as in tumor tissue (pathological conditions). However, although numerous proteomic studies have been carried out on tumor tissue and body fluids from different cancer types and many candidate proteins have been identified as potential cancer biomarkers, relatively few have finally received FDA approval. More recently, tumor immune cell infiltration or immune profiling using RNA/protein biomarkers have proved to be useful as prognostic biomarkers or as predictive biomarkers for selecting patients that could benefit from immunotherapy (see Section 5.5). Anti-programmed cell death-1/programmed cell death-ligand 1 (PD1/PD-L1) antibody is approved for the first or second line of treatment for various cancers. For example, in gastroesophageal cancers, a combined positive score for microsatellite instability or mismatch repair, tumor mutational burden, and PD-L1 expression is used for immunotherapy selection [82].

Proteins from tumor tissues are mainly studied by immunohistochemistry (IHC), while enzyme-linked immunosorbent assay (ELISA) is commonly used for body fluid protein biomarkers. Proteomic technologies applied for liquid biopsies in cancer diagnosis are reviewed in Ding et al. [83]. Moreover, glycomic profiling can be performed by mass spectrometry, but lectin microarrays are increasingly being used [84].

### 2.6. Cellular Phenotype

Changes in gene and protein expression can result in changes in morphology and function of cells, which is known as cellular phenotype. These phenotypic features are reflecting the variety of pathways involved in the expression of that particular phenotype. Therefore, cellular responses, such as DNA damage response (including DNA repair), levels of oxidative stress, or cell death (apoptosis), among others, can be used as cellular biomarkers for cancer. Notably, if the phenotypic assay used to analyze the cellular biomarker is high throughput, it may be easier to implement in a clinical setting than gene or protein assays.

Since many cancers are due to genomic instability, which can be related to alterations in DNA damage response pathways, this can be a sign of tumor growth and progression. Moreover, genomic instability and DNA damage response alterations are potential biomarkers of success for new immunotherapy drugs [85] (see Section 5.5). Finally, as many cancer treatments are targeting DNA damage response pathways, alterations of these pathways can be a biomarker of response to cancer treatment.

A recent meta-analysis of 55 case-control studies evaluated the association between DNA repair phenotype and risk for 12 different cancers [86]. According to this study, individuals with lower DNA repair capacity have a higher risk of developing cancer. Moreover, these results were obtained for all studied cancer types, suggesting that the DNA repair phenotype is a good candidate to be used as a cancer biomarker.

Another example of a phenotype assay would be the analysis of levels of cellular oxidative stress in body fluids as a biomarker of response to cancer treatment. One of the best methods to assess oxidative stress is to study levels of 8-hydroxydeoxyguanosine (8-oxo-dG). Interestingly, several studies have observed that levels of 8-oxo-dG in urine or blood may be a useful biomarker of therapy response in cancer patients [87,88,89].

In addition, phenotypic cellular apoptosis measured in blood after in vitro irradiation has been used as a predictor of radiation-induced cancer [90] and as a biomarker of late radiotherapy toxicity in breast cancer patients (see Section 5.5).

## 3. Sources of Molecular Cancer Biomarkers

Cancer biomarkers can be studied from a variety of sample types, with the tumor tissue being the most widely analyzed so far. An alternative to tumor biopsies is liquid biopsies, which are predominantly non-invasive. The most common non-tumor sample types used for cancer biomarker analysis are blood, urine, stool, and, less commonly, exhaled breath, saliva/buccal swabs, cerebrospinal fluid, sputum, and other body fluids (Table 2).

### 3.1. Blood

Different parts of the blood, such as white blood cells (WBCs), circulating tumor cells (CTCs), plasma, serum, or EV can be used for biomarker testing.

#### 3.1.1. WBCs

DNA from WBCs is used for germline genetic variant detection; for example, BRACAnalysis CDx (Myriad Genetics, Inc., Salt Lake City, UT, USA) is used to screen for changes in coding regions of *BRCA1* and *BRCA2* genes. The germline genetic variant detection is performed by using PCR and Sanger sequencing/NGS for SNV and indel detection and by using multiplex PCR for large deletions and duplications in precision medicine for breast, ovarian, pancreatic, and prostate cancer.

In addition, for hematological cancers, such as leukemias, lymphomas, or myelomas, WBCs are the primary specimen for diagnosis, from basic morphology studies to immunophenotyping, using panels of fluorochrome-labeled antibodies to analyze antigen expression by flow cytometry. Moreover, in these types of cancer, WBCs are used in conventional cytogenetics/molecular cytogenetics/FISH to detect mostly aneuploidy or translocations; in addition, WBC DNA is used in molecular genetic tests to detect sequence mutations. These WBC-based biomarkers are useful in diagnosis, prognosis, detection of residual disease, and prediction of response to treatment or relapse.

#### 3.1.2. CTCs

Some cancer cells are shed in circulation by solid tumors and have a promising future in liquid-biopsy-based cancer diagnostics and monitoring [92]. The biggest hurdle in their detection is their extremely low number, of approximately 1 CTC/mL in blood. Despite the low number, technical improvements have made it possible to capture, identify, and count them. The detection methods include those based on their physical characteristics, such as large cell size, electrical properties (e.g., CTCs can have a more negative charge compared to leukocytes [93]), or immunological properties, which can help to identify surface proteins on CTCs. Currently, nanomaterials are being tested to increase the efficiency of immuno-capture and detection [94]. As their number is increased in metastatic disease, they have use in monitoring metastatic breast cancer, CRC, and prostate cancer.

#### 3.1.3. Plasma/Serum

Tumor-associated ctDNA, ctRNA, and protein changes can also be observed in plasma or serum, and nowadays plasma-based cancer diagnosis is at the forefront of cancer biomarker development. Protein biomarkers have been widely used for decades for cancer diagnosis and monitoring from plasma samples, and some examples are mentioned in Section 5.2 and Section 5.4.

Moreover, a few NGS panels for detecting cancer-associated genetic alterations in plasma/serum are now available and approved for use in the clinic. For example, FoundationOne Liquid CDx (Foundation Medicine, Inc., Beverly, MA, USA) uses targeted high-throughput hybridization-based capture technology and detects mutations in 311 genes, including four gene rearrangements and CNAs in three genes, in ctDNA from plasma samples for use as companion diagnostics for targeted therapies. Another similar NGS-based plasma ctDNA test approved is Guardant360 CDx (Guardant Health, Inc., Palo Alto, CA, USA), which detects mutations in fifty-five genes, fusions in four genes, and CNAs in two genes, and is used as a companion diagnostics for lung cancer. Other plasma-based tests approved for clinics are mentioned next. The Therascreen PIK3CA RGQ PCR Kit (Qiagen Gmbh, Hilden, Germany), which is based on real-time qualitative PCR, is used for the detection of 11 mutations in the *PIK3CA* gene in FFPE tumor DNA or ctDNA of patients with breast cancer to identify patients for targeted treatment with PIQRAY^®^ (Alpelisib). Epi proColon (Epigenomics AG, Berlin, Germany) is used for screening CRC in individuals older than 50 years and that cannot be screened by standard methods. It detects methylation in the promoter region of the Septin 9 gene from plasma DNA, using real-time methylation-specific PCR. Finally, COBAS EGFR mutation test V2 (Roche Molecular Systems, Indianapolis, IN, USA) detects somatic mutations in the *EGFR* gene from FFPE tumor DNA or plasma ctDNA and is used as a companion diagnostics method for the selection of targeted therapies in lung cancer patients [95]. It is noteworthy that the main limitation of plasma-based tests is the low concentration of tumor-associated biomolecules present in this fluid, and most NGS-based tests are, therefore, approved as single-site assays carried out at specific laboratories.

### 3.2. Urine

Urine is mainly used for biomarker testing in bladder or prostate cancer. An in vitro RNA-based assay (Progensa PCA3 assay, from Hologic, Inc., Marlborough, MA, USA), which calculates the ratio of *PCA3*/*PSA* RNA molecules (PCA3 score), is approved for urine samples collected after digital rectal exam to aid in the decision for prostate biopsy (score of <25 is associated with a low probability of positive prostate biopsy). Another urine-based diagnostic test for suspected bladder cancer is the Urovision Bladder Cancer kit, which detects aneuploidies for chromosomes 3, 7, and 17 and loss of 9p21 locus by FISH. The test is used for initial diagnosis and tumor recurrence monitoring. Finally, a simple protein-based diagnostic and monitoring test for bladder cancer, the Alere Nmp22 Bladderchek test, is an enzyme immunoassay-based quantitative testing of nuclear matrix protein (NMP22) in urine samples for bladder cancer diagnosis.

### 3.3. Stool

Stool samples are mainly used for CRC screening; these tests are simple to perform, and some of them can even be performed at home. The basic test for CRC screening is fecal immunochemical testing, which detects blood released in small amounts by tumors and polyps into the stool. However, the test is non-specific, as other conditions such as hemorrhoids can also give positive results. On the other hand, CRC-related gene mutations can also be detected in the stool DNA of patients. We detected gene mutations in stool DNA of CRC patients, using a targeted amplicon gene panel by NGS; these mutations correlated to those seen in matched FFPE tumor tissues [14]. Interestingly, a CRC screening test, Cologuard (Exact Sciences, Inc., Marlborough, MA, USA), which is based on detecting CRC-associated DNA mutations in stool samples, is approved by FDA. It has a sensitivity of 92% for CRC detection and 42% for large polyps, compared to 74% and 24%, respectively, when using fecal immunochemical testing.

Stool samples are also useful for microbiota profiling, which has also gained relevance as a biomarker, not only for CRC but also for other gastrointestinal cancers, including pancreatic cancer [96,97]. Moreover, the bacterial profile can also predict response to cancer therapy.

### 3.4. Exhaled Breath

Cancer gene mutations can also be detected from the exhaled breath of lung cancer patients (extensively reviewed in Reference [12]).

### 3.5. Saliva/Buccal Swabs

The main advantage of saliva/buccal swabs as a source of cancer biomarkers is their ease of collection. DNA isolated from saliva/buccal swabs is mostly used for genotyping germline genetic variants, e.g., *TMPT* variant detection for risk evaluation before treatment with thiopurines in leukemias. Moreover, and similarly to plasma samples, saliva is also a source of tumor-associated nucleic acids, proteins, metabolites, and EVS to be used as cancer biomarkers, for example, in oral cancer or head and neck cancers [98]. In this regard, two meta-analyses have shown hypermethylation biomarkers with high specificity [99] and IL1β, IL6, and IL8 as early interleukin biomarkers [100] in saliva samples for diagnosis and early detection of oral cancer. In addition, changes in the oral microbiome can also be useful biomarkers not only in head and neck cancer [98] but also in pancreatic cancer [96]. Finally, human papillomavirus DNA in saliva can be a useful biomarker for treatment response and recurrence in human papillomavirus-associated head and neck cancers [101].

### 3.6. EVs

EVs are small lipid-bound vesicles released by all cells that contain different types of biomolecules, such as proteins and nucleic acids, and that play an important role in intercellular communication. Depending upon their origin, they can be grouped into exosomes (endocytic origin), microvesicles (formed from plasma membrane budding), or apoptotic bodies. The content or the type of cargo they carry depend on their cell of origin, and, thus, their analysis in body fluids can contribute to identifying new cancer biomarkers. Differential expression of membrane-bound and intra-vesicular proteins and miRNAs of EVs has been reported in plasma, urine, or other body fluids of cancer patients compared to healthy individuals (reviewed in Reference [102]). Moreover, in addition to protein and miRNAs, DNA alterations, such as cancer gene mutations can also be detected in EVs of cancer patients [103].

However, the limitation of these EV-associated biomarkers is that EVs are heterogeneous vesicles with a range of different sizes and types, and, therefore, the results can vary depending upon the isolation method or kits used. The methods generally applied are based on density-gradient ultracentrifugation, filtration/size exclusion chromatography, EV-precipitation, affinity interactions (by antibodies, lipid-binding proteins, and lectins), or microfluidic separation (based on immunoaffinity, microporous filtration, acoustic nanofiltration, and porous micropillars) [104]. Notably, miRNAs are found to be more concentrated in serum EVs than as free circulating serum molecules, and their EV concentration is found to increase with increasing malignancy in cervical cancer and better discriminate cancer from controls [27]. A manually curated database named EVAtlas, created by Liu et al. [105], provides a comprehensive compilation of ncRNA expression in EVs from 2030 small sequencing datasets. Using the dataset, the authors identified *miR-451a* as a potential lung cancer biomarker in plasma EVs [105]; however, they observed that miRNA EV expression does not necessarily correlate with tumor expression. *MiR-451a* was found to be highly expressed in plasma EVs of lung cancer patients compared to controls, while its expression was lower in lung tumor tissue compared to normal lung tissue. The authors postulated that *miR-451*, which acts as a tumor suppressor and is expressed in normal lung tissue, might be packed in EVs and removed from tumor cells.

## 4. Techniques Used to Detect Molecular Cancer Biomarkers

### 4.1. FISH

It employs a fluorescently labeled probe that hybridizes with DNA to detect gene copy number changes (e.g., *HER2* amplification) or gene fusions in tumor sections or cells (Figure 1a). Some variants of FISH include multiplex FISH, spectral karyotyping, and comparative genomic hybridization. Spectral karyotyping is a 24-color chromosome painting assay, which detects chromosomal abnormalities with high sensitivity. It can be used to detect chromosomal biomarkers of cancer diagnosis and prognosis, especially in hematological malignancies, sarcomas, carcinomas, and brain tumors [106].

### 4.2. PCR/Real-Time PCR/Digital PCR

PCR-based targeted genetic profiling is the most common technology used in cancer diagnostics for both DNA- and RNA-based applications. It is used for the detection of small DNA mutations (e.g., *EGFR* mutations), gene fusions (e.g., RNA-based testing for *ALK*), or DNA methylation analysis using methylation-specific PCR (e.g., *MGMT* promoter methylation in glioblastoma or Septin9 gene methylation in CRC). Many modifications of this basic method are continuously being developed to increase the sensitivity of detecting biomarkers from trace sources.

### 4.3. NGS

NGS is finding application in genetic screening of both germline variants and somatic mutations, including SNVs, indels, and CNAs. It is also being used for RNA-based biomarkers, such as gene fusions and RNA sequencing. The approaches include both amplicon-based screening using primer panels to amplify regions of interest harboring driver gene mutations, or targeted capture and hybridization for selecting fragments of interest for sequencing using capture probes. Different kinds of NGS gene panels have been developed: cancer-specific panels (e.g., for lung cancer, CRC, and breast cancer), general pan-cancer panels for solid tumors or hematological cancers, or panels designed to detect genomic changes for targeted therapies. Noteworthy, NGS-based tests are sensitive to the platform and methods used, and they are therefore mainly approved for testing at a specific site. Other challenges in NGS-based methods relate to differentiating cancer driver mutations from passenger mutations and setting a minimum threshold mutant allele frequency for variant calling.

Nowadays, new machine learning and computation methods are being developed to relate NGS mutations to clinical significance or therapy response. One such approach, named TARGETS (TreAtment Response Generalized Elastic-neT Signatures), is shown to predict the response to specific drugs, based on NGS DNA/RNA profiles [107].

### 4.4. Flow Cytometry

It is often applied in leukemia and lymphoma diagnostics to identify and count cells by using a panel of fluorescently labeled antibodies. It is also deployed to quantitate DNA in cancer cells by treating them with DNA-binding, light-sensitive dyes. Changes in DNA quantity indicate cancer recurrence in breast, prostate, or bladder cancer. What is more, it has application in CTC-based biomarkers, as well.

### 4.5. Gene Expression Microarrays

They are used to study differentially expressed genes in tumor samples and to classify tumors into molecular subtypes, both of which can be predictive of prognosis or treatment response. For example, MammaPrint, a microarray-based prognostic test, uses a 70-gene expression profile from FFPE tissue to predict early-stage breast cancer patients with a high/low risk of recurrence. The implementation of such microarray-based molecular classifications of breast cancer by MammaPrint, TargetPrint, or BluePrint has shown that these tests are useful for better management of this cancer [108]. Other gene-expression-based tests, such as Oncotype DX, have applications as predictive biomarkers of chemotherapy response and cancer prognosis (see Section 5.5).

### 4.6. IHC

IHC is a routinely used method in cancer diagnostics/pathology for detecting proteins expressed by cancer cells in tumor tissues. Developments in this technology include (i) multiplex IHC, with cycles of antibody staining, imaging, and quenching, which can be repeated by using different antibodies on the same tissue section; and (ii) other technologies, such as tyramide signal amplification, MultiOmyx^TM^, and fluorescent quantum dot nanocrystals, with more sensitivity for detecting low-abundance proteins and with a high signal-to-noise ratio.

### 4.7. ELISA

It is the most commonly used protein-analysis method in clinical practice, especially in body fluids. New developments, such as electrochemical ELISA assays, increase the sensitivity of ELISA for protein biomarkers at low concentrations in body fluids by amplifying the signal. They are more cost-effective and easier to use [109].

### 4.8. Lectin Microarrays

Lectin microarrays are used for high-throughput profiling of glycans, especially for studying differences in glycomic profiles between cancer and normal tissue or for identifying new biomarkers in plasma or EVs [110].

### 4.9. Other Proteomic Tools

Mass spectrometry (MS) and reverse-phase protein arrays (RPPA) are two other proteomic tools that can be used to detect a large number of proteins in cancer samples. MS can be used either as a global profiling tool for cancer biomarker discovery or as a targeted approach (reviewed in Reference [111]), whereas RPPA is a targeted antibody-based proteomics platform. Sometimes, RPPA is utilized to validate candidate protein biomarkers detected by MS profiling. Both can detect and quantify proteins and their post-translational modifications in tumor tissues or biological fluids. RPPA has a higher throughput and lower cost than MS; moreover, it has a lower limit of detection and higher sensitivity. A recently optimized RPPA platform analyzed 240 validated antibodies and detected important proteins in cancer [112].

### 4.10. Biosensors/Nanotechnology

The biggest hurdle in the cancer biomarker field is the very low concentration of analytes in non-tumor tissue samples, such as blood or other body fluids. The use of biosensors and nanotechnology is being tested to increase the sensitivity and specificity of detection. Biosensors are devices that detect a biomarker by a chemical process which is converted into an electric signal by a transducer, and the signal is then processed and amplified [113]. Moreover, gold nanoparticles, quantum dots, nanotubes, and nanoribbons provide a high surface-to-volume ratio, allowing different molecules (antibodies, linkers, small molecules, etc.) to be densely attached to their surface, thereby increasing the sensitivity of detection by biosensors. They can be applied to capture and detect some cancer biomarkers, such as ctDNA/RNA/miRNAs (e.g., *miR-141* in serum of prostate cancer patients [114], or DNA methylation in ctDNA for cancer detection [115]), proteins, CTCs, and EVs in body fluids [116]. Some of the proteins expressed by CTCs and used for detecting these cells by nanotechnology include EpCAM, PTK7, HER2, and Cd2/Cd3. Moreover, although not yet in clinical use, some nanoribbon sensor chips can detect circRNA in gliomas [117] or *miR-17-3p* in CRC [118], employing oligonucleotide molecular probes complementary to the target sequence.

### 4.11. Microfluidics

Microfluidic chips are being developed in combination with other biomarker detection techniques for use in clinical applications (reviewed in Reference [119]). For example, microfluidic chips have been optimized to detect cancer-associated proteins in oral cancer [120] or to capture CTCs by using new methods combining cell size and immunoaffinity in prostate cancer [93]. Moreover, Chu et al. [116] developed a nanomaterial-based microfluidic chip for ultra-sensitive detection of miRNAs at attomole levels for use in cancer diagnosis. Similarly, microfluidic chips combined with digital PCR have also been developed to analyze ncRNA or DNA methylation from liquid biopsy [121].

### 4.12. CRISPR-Based ctDNA/RNA Detection

Another promising technique that could simplify the detection of ctDNA/RNA and increase its sensitivity and specificity has been recently developed based on clustered regularly interspaced short palindromic repeats (CRISPR) technology. It is known that different CRISPR-associated (Cas) enzymes can be used to detect different nucleic acids. Therefore, by combining an RNA-guided RNase Cas13a, a target-specific CRISPR RNA, and a labeled reporter RNA, RNA signals can be detected without the need for nucleic acid amplification steps. Interestingly, the CRISPR/Cas13a system integrated into microfluidic chips with biosensors has been successful in detecting miRNA biomarkers in serum samples of brain cancer patients with remarkable sensitivity of detection (10 pM in a volume of less than 0.6 µL and in less than 4 h of processing time) [122]. Similarly, an assay using the CRISPR-Cas14a system and strand displacement amplification for detecting *miR-21* expression in blood was shown to discriminate cholangiocarcinoma patients from controls [123].

### 4.13. Synthetic Biomarker Technology

To overcome some of the challenges associated with cancer biomarkers, such as low sensitivity or specificity and technical limitations, a new class of synthetic biomarkers is being developed (reviewed in Reference [124]). The theory behind activity-based synthetic biomarkers is to administer an exogenous agent that includes a bioengineered sensor component. This agent is targeted to specific activity/physiology characteristics of cancer cells and leads the tumor to shed synthetic biomarkers, resulting in the production of a signal that can be detected. Examples include proteases-activated synthetic biomarkers and small-molecule probes.

A summary of technologies, their applications, and their advantages and disadvantages is presented in Table 3.

## 5. Clinical Applications of Cancer Biomarkers: Examples

The clinical applications of cancer biomarkers are extensive, and their ultimate goal is to achieve precision medicine to optimize prevention, screening, and treatment strategies of cancer. These applications include risk assessment; screening and early detection; accurate diagnosis; patient prognosis; prediction of response to therapy; and cancer surveillance and monitoring response.

Updated lists of biomarkers currently being used in cancer patients can be found in the National Comprehensive Cancer Network Compendium [125] and the Table of Pharmacogenomics Biomarkers in Drug Labelling from the FDA (therapeutic area = oncology) [126], as well as in the Tumor Marker List from National Cancer Institute [91] (Table 2). Moreover, a knowledge base, OncoMX, has recently been developed for exploring cancer biomarkers in the context of related evidence [127].

### 5.1. Cancer Risk Assessment Biomarkers

A cancer risk or susceptibility biomarker is used to identify individuals with a higher probability to develop cancer compared to the standard risk in the general population. Cancer risk biomarker tests include DNA repair phenotype assays, as well as genotyping assays for germline variants. DNA repair has shown clear interindividual variability related to cancer susceptibility [128]. Several technologies have been reported to quantify DNA repair capacity (and also DNA damage and DNA damage response): comet assay, ɣH2AX foci formation, host cell reactivation assay, DNA repair beacons, and others (reviewed in Reference [128]). However, in the last decades, genotyping assays have gained importance thanks to the development of high-throughput NGS tools (see Section 2.1). In a recent study performed with a large multicenter cohort, the personal risk for hereditary cancer syndromes, among other disorders, was evaluated in healthy individuals [129]. Disease-predisposing variants related to cancer syndromes were present in 7.7% of individuals analyzed. Clinically significant variants were commonly detected in *MUTYH*, *CHEK2*, *APC*, *ATM*, *BRCA1*, *BRCA2*, *MITF*, *HOXB13*, *PMS2*, *PALB2*, *NBN*, *BRIP1*, *MSH6*, *SDHA*, and *BARD1*. These findings would prove the utility of using genetic screening as part of regular medical care [129], although there are doubts about the interpretation of variants of uncertain effect [130].

Once a biomarker of cancer risk is identified and validated, it should be incorporated into a comprehensive risk assessment model, which should include also other risk factors, such as environment and lifestyle. Individuals identified to be at high risk of developing cancer could engage in changes in their lifestyle and could benefit from enhanced surveillance, prophylactic surgery, and/or some sort of chemoprevention.

### 5.2. Screening and Early Cancer Detection Biomarkers

The purpose of these biomarkers is to detect cancer in otherwise healthy patients, and without having shown any signs of disease. Actually, they are suggesting the presence of cancer, which has to be diagnosed with other medical approaches. The main justification for their use is the fact that if cancer is detected in an early and asymptomatic stage, the survival rate increases, and the probabilities of complications or morbidities decrease. However, in some cases, the use of these biomarkers leads to overdiagnosis, that is, detection of a cancer that would never cause any symptoms.

A good screening biomarker assay must be highly specific, i.e., with a very low false-positive rate, and, ideally, it should be also noninvasive and inexpensive. Some of the problems caused by overdiagnosis and false-positive rate are further invasive medical procedures, patient psychological distress, and high costs for the healthcare system, which are otherwise unnecessary.

Several blood-based screening biomarkers have been used or are currently used in clinical practice, e.g., alpha-fetoprotein for liver cancer, PSA for prostate cancer, CA19-9 for pancreatic cancer, and CA125 for ovarian cancer, among others [131]. However, some of these biomarkers do not meet the requirements of high specificity and sensitivity; for example, this is the case for blood PSA screening for prostate cancer. Furthermore, this biomarker does not allow us to distinguish individuals with benign prostatic hyperplasia from those with malignant prostate cancer. Therefore, the current prostate cancer screening program using PSA would not be desirable in a clinical setting, and it would only be recommended for men who express a specific interest in screening [132]. New tests are under evaluation, such as a recently developed biomarker panel combining filamin-A gene, age, and prostate volume which has a better performance than PSA alone, especially in men with benign hyperplasia [133].

Interestingly, promising multi-cancer early detection tests on liquid biopsies are now being designed to complement single-cancer screening tests, and they seem to perform better [134,135,136]. However, concerns have risen about their clinical utility, as well as about overdiagnosis, overtreatment, and the accuracy of identification of tissue of origin [137].

### 5.3. Accurate Cancer Diagnosis Biomarkers

Diagnostic biomarkers are used to confirm the presence of cancer or to identify a subtype of cancer. The usefulness of these biomarkers is that proper diagnosis can lead to proper treatment and, therefore, best chances of survival. Some screening biomarkers are also used as diagnostic biomarkers; however, the latter is only applied to symptomatic patients, whereas screening biomarkers are applied to asymptomatic individuals. Despite diagnostic biomarkers can help to detect cancer or to classify patients into subtypes, they are not sufficient for a final diagnosis and need to be combined with other diagnostic procedures, such as imaging or biopsies.

### 5.4. Patient Prognosis Biomarker

Once a tumor has been diagnosed, a prognostic biomarker provides information about the probable course of the disease, including its recurrence, progression, and patient’s overall survival, regardless of the treatment. Sometimes, these biomarkers can reflect tumor burden, and then they can help in determining the stage of cancer (e.g., the tumor–node–metastasis classification). Some examples of prognostic biomarkers widely used are protein biomarkers, such as CEA for CRC, CA19-9 for pancreatic cancer, and CA125 for ovarian cancer; and some tests based on gene expression signatures for breast cancer, such as MammaPrint and Prosigna [125,134,138,139]. Other examples would be the genetic alterations that facilitate an accurate risk-stratification of patients with acute leukemia that are associated with patient outcomes [140,141].

The information obtained with these biomarkers can be useful for clinicians to make decisions for aggressive or prolonged treatments. However, some of the currently available prognostic biomarkers have also been designed to be predictive of chemotherapy benefits (see below), as this is preferable in a clinical setting.

### 5.5. Biomarkers Predicting Response to Cancer Therapy

It is well-known that treatment decisions are critical in cancer patient management, and often there is uncertainty in the levels of response, as well as lack of precision, side effects, unnecessary overtreatment, etc. However, considerable progress is being made and predictive biomarkers are increasingly playing a key role in the optimization of cancer treatment, based on the idea that specific tumor alterations or specific germline genetic variants (pharmacogenetics) yield a certain pattern of sensitivity to cancer therapy agents.

Predictive biomarkers aim to estimate the effect of a specific therapy on a cancer patient before treatment has started. According to the results of the biomarker assay, cancer patients can be classified as probable responders or non-responders to a specific therapy, either chemotherapy, endocrine therapy, radiotherapy, or the emerging targeted strategies and immunotherapy. Some of these biomarkers can also identify those patients that will likely show severe toxicity after therapy. Predictive biomarkers can be very useful for adjusting treatment doses or guiding alternative therapies in patients classified as non-responders or with a high risk of toxicity.

Biomarkers to predict tumor response to classical chemotherapy or endocrine therapy are few, despite being the most extensively used to treat cancer patients [142,143]. Traditional examples of cancer predictive biomarkers are pharmacogenetic-based ones, such as germline variants on *TPMT* or *TYMS* genes that estimate the effectiveness/toxicity of treatment with mercaptopurine for leukemia or with fluorouracil for colon, bladder, and gastric carcinoma, respectively (reviewed by Reference [144]). Moreover, somatic cancer mutations can also help to predict tumor drug response. In this regard, data related to the sensitivity of 1000 human cancer cell lines to different drugs is compiled in the database Genomics of Drug sensitivity in Cancer (www.cancerrxgene.org, accessed on 1 March 2022). More information about currently used and potential future biomarkers of this kind can be found at the Pharmacogenomics Knowledgebase [145]. Recently, multi-gene predictive biomarkers have been developed, such as the Oncotype DX Breast Recurrence Score test, which measures the expression of 21 genes on breast cancer samples. This test allows clinicians to select the therapy that will be optimal for women with hormone receptor+ and HER2− early stage invasive breast cancer according to their score (prognostic biomarker): either chemotherapy plus endocrine therapy or endocrine therapy alone [146,147]. Additionally, the test gives information about distant recurrence (predictive biomarker), and it has been incorporated by the American Joint Committee on Cancer into the breast cancer staging system [148].

Radiotherapy effects on cancer patients vary greatly, even in patients with similar tumor types and treated with similar radiation schemes, both in terms of tumoral response and of early or late adverse reactions in non-tumoral tissues [149]. Several biomarkers to assess tumor radiosensitivity have been studied, including tumor molecular signatures, expression of specific mRNA molecules or proteins, mutations at specific genes involved in DNA repair, or the less studied EVs and CTCs [150,151,152]. Concerning biomarkers aimed to predict the risk of radiation-induced toxicity in normal tissues, the research has focused on the assessment of DNA damage response (comet assay, ɣH2AX foci formation, or micronucleus formation) and, more recently, on apoptosis, all of which are studied after in vitro irradiation of peripheral blood lymphocytes from patients [151,153]. Other well-studied predictive biomarkers of toxicity are germline genetic variants from patients with breast cancer, prostate cancer, and other cancers [154]. In addition, protein biomarkers present in blood from breast cancer patients have been related to cardiotoxicity after radiotherapy [151,155].

In the case of targeted cancer treatments, first, the alteration that is driving tumor growth in that particular cancer is identified. Then a treatment strategy is developed to specifically target that alteration. Therefore, targeted therapies only work in a subset of cancers, those that have the specific alteration for which the therapy was designed. It is important, if not essential, to perform a biomarker assay to identify those individuals who will benefit from therapy in order to increase efficacy and diminish costs [142]. These kinds of assays are often called companion diagnostics and are usually approved by the regulatory agencies in conjunction with the drug they are paired with. The information provided by the companion diagnostic tool is essential for the safe and effective use of the therapeutic product [156]. An example is an immunohistochemistry test for increased expression of HER2 receptor to select patients that would benefit from therapy with trastuzumab, an anti-HER2-targeted agent used to treat breast, gastric, and gastroesophageal cancers [157]. Another example is EGFR therapies, mainly used in metastatic NSCLC, and which target cancerous cells with *EGFR* mutations at exon 19 or 21. More recently, NGS-based companion diagnostics have been developed to detect cancer-associated genetic alterations in plasma/serum ctDNA (see Section 3.1.3).

Moreover, some cancer biomarkers help in the assessment of the risks and benefits of a particular drug, even though the information provided by these biomarkers is not required for the use of that drug. This kind of assay is referred to as complementary diagnostics since it provides additional information to the physicians [156]. For example, the FoundationFocus CDx BRCA loss of heterozygosity (LOH) assay is an FDA-approved complementary diagnostic test for rucaparib, a poly (ADP-ribose) polymerase inhibitor used to treat recurrent ovarian carcinoma. While rucaparib improves the progression-free survival rate in patients with high genomic LOH, those with lower genomic LOH may also benefit [158].

In the last decade, immunotherapy, especially immune checkpoint inhibitors, have demonstrated high efficacy against some cancers by enhancing anti-tumoral immunity, while many others do not respond or even show serious side effects. The discovery of biomarkers to predict sensitive or refractory tumors to this kind of therapy is urgently needed. Moreover, there is a need for optimization of the currently FDA-approved biomarkers of response to immunotherapy, including expression of PD-L1, microsatellite instability, and tumor mutational burden [159,160]. Of these, PD-L1 is the most commonly used in clinical practice, especially in NSCLC. Cancer cells that express PD-L1 can attenuate or inhibit the activity of tumor-infiltrating lymphocytes, which express the receptor of PD-L1. This is a mechanism used by tumor cells to escape immune surveillance. The blockade of this interaction by using antibodies, either against PD-L1 or its receptor, makes lymphocytes reactivate and enhance their antitumoral effect. Therefore, tumors overexpressing PD-L1 are more likely to respond to these antibodies, even though those with lower PD-L1 expression may also benefit. However, the accuracy and clinical utility of this biomarker need to be improved [159,160].

A good summary of key cancer predictive biomarkers clinically adopted, as well as those showing potential for clinical translation, can be found in Reference [142].

### 5.6. Biomarkers for Cancer Surveillance and Monitoring Response

A monitoring biomarker is assessed serially over time, e.g., during treatment with curative intent or after it has finished. This can allow for comparisons to observe, for example, real-time overall disease burden, to detect worsening of the disease, or to follow disease response to treatment.

Liquid-biopsy-based biomarkers are the best option for minimal residual disease monitoring and cancer surveillance. In this sense, several studies have reported ctDNA as a promising good monitoring biomarker, since it is believed that ctDNA levels correlate with tumor burden over time. Therefore, monitoring ctDNA in cancer patients could help to detect early recurrences or residual cancer that would otherwise remain undetected by other methods, such as imaging [161]. Other monitoring biomarkers are blood proteins (e.g., CEA, CA19-9…), although they have some disadvantages compared to ctDNA, such as lower tumor-specificity and longer half-life. As a recent example, driver mutations in ctDNA (*EGFR*, KRAS, or *BRAF*) and serum concentrations of Cyfra21-1, and possibly CA125, have been described as relevant useful biomarkers for therapy response monitoring and early detection of progression during therapy in lung cancer [162]. However, clinical implementation of monitoring biomarkers is challenging, as there are still some methodological and biological limitations [163].

## 6. Steps in the Search for New Biomarkers

**Figure 2:**
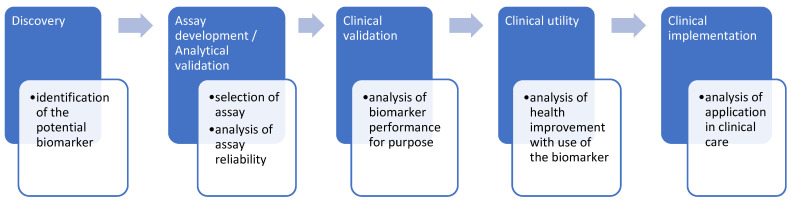
Schematic steps on the search for new biomarkers.

Even though considerable progress has been made, there is an urgent need for the discovery and development of new effective biomarkers in the field of oncology. The steps involved in the pipeline of development of cancer biomarkers are as follows: discovery, assay development/analytical validation, clinical validation, clinical utility, and finally clinical implementation [164,165,166] (Figure 2).

### 6.1. Discovery

It is the initial step based on the identification, selection, and prioritization of potential individual or a group of biomarkers (biomarker signature) through exploratory preclinical studies. Ideally, before starting, researchers should clearly define the purpose of the biomarker and the specific clinical context [164,167].

The advent of new techniques, such as NGS, gene expression arrays, protein MS, and other high-throughput technologies, has provided researchers with an enormous amount of data in a short time and at a low cost, which has sometimes led to the generation of data-driven hypotheses [168]. However, a correct study design and proper data analysis must be employed to be able to select relevant data from which reliable candidate biomarkers can be identified.

A correct study design includes, among other things, a good selection of the target population, enough statistical power, consideration of possible confounding factors, and randomization and blinding to avoid bias [167]. Later, appropriate data analysis is equally important, as it can influence the reproducibility and robustness of results. For example, careful handling of missing data should be implemented, as well as statistical correction of multiple comparisons. The latter is especially useful when analyzing a group of biomarkers, which usually perform better than individual ones [167,169].

### 6.2. Assay Development and Analytical Validation

After a potential cancer biomarker (or a biomarker signature) has been identified, an assay is developed to detect or quantify the biomarker in a patient specimen. A technical protocol must be specified, including sample collection, processing, and storage procedures [170].

The next step is to establish whether the selected biomarker assay can detect or measure what it is intended to detect or measure, which is called analytical validation. It is defined as a process to establish that the performance characteristics of the assay are acceptable in terms of its analytical sensitivity, specificity, accuracy, and precision, which includes repeatability and reproducibility [170]. Definitions of these terms are shown in Table 4.

### 6.3. Clinical Validation

Clinical validation is defined as a process to establish that the biomarker (through its assay) can acceptably identify, measure, or predict the relevant clinical concept [170]; that is, the assay reliably divides the population of interest into two or more groups of individuals that have significant differences [173]. There is not necessarily evidence that the biomarker improves clinical care [174].

The performance of the biomarker is estimated in terms of diagnostic sensitivity, diagnostic specificity, positive predictive value (PPV) and negative predictive value (NPV), receiver operating characteristics (ROC) curve, and area under the ROC curve (AUC^ROC^) (Table 5). Diagnostic sensitivity and specificity terms are distinct from the previously mentioned analytical terms, e.g., high analytical sensitivity does not necessarily correlate with acceptable diagnostic sensitivity [172]. It is generally accepted that a good biomarker should have values of diagnostic sensitivity and specificity of at least 90% and AUC values higher than 75% (values of AUC above 90% would correspond to an excellent biomarker) [175,176]. However, for clinicians, PPV and NPV represent more interesting probabilities than diagnostic sensitivity and specificity. PPV is the proportion of individuals that are positive among all the test-positive individuals, and NPV is the proportion of individuals that are negative among all the test-negative individuals.

Clinical validation of a cancer biomarker usually needs external validation with an independent set of samples, entirely different from the one used in the discovery step. This can be performed either in a retrospective or prospective study [164,167].

### 6.4. Clinical Utility

A cancer biomarker must have high levels of evidence of clinical utility, besides analytical and clinical validity, if it is meant to be applied to routine practice to guide healthcare. The clinical utility of a cancer biomarker is the demonstration that the use of the biomarker in an individual will lead to a net improvement in his/her health outcome (or to a decrease in treatment toxicity or healthcare costs) compared with that of an individual whose care is managed without the use of that biomarker [171,173]. Therefore, the outcome benefit of using the biomarker must be clinically and/or financially meaningful. Factors to be considered to determine the clinical utility of a cancer biomarker are extensively addressed in the [173] review. It is important to assess both the effectiveness of the biomarker and the benefit-to-risk ratio.

The assessment of clinical utility usually requires a prospective clinical trial or a prospective–retrospective study with a completely independent dataset and performing a controlled comparison with standard options of clinical management [173].

### 6.5. Clinical Implementation

Some key aspects of clinical implementation of a cancer biomarker are regulatory approval by authorities (e.g., FDA or European Medicines Agency), commercialization, health insurance coverage, and incorporation into clinical practice guidelines [164]. These factors are highly dependent on each national health system and particular national regulations. Moreover, regulatory processes may be different if the biomarker assay is an in vitro diagnostic device or a laboratory-developed test [164]. Finally, acceptability to physicians and patients is also essential when implementing a biomarker assay in the clinic. Moreover, healthcare providers should know what biomarker assay to use, when, and how to interpret the results to take advantage of the full potential of the biomarker. Therefore, good clinical guidelines are critical and must be updated regularly. Equally important is the need that the healthcare facilities are equipped with the appropriate infrastructure for efficient sample collection and handling, as well as testing, if no subcontract is applied.

Big challenges remain in the process of cancer biomarker development, especially the need to generate high levels of evidence of a cancer biomarker value. Recently, Parker et al. [177] performed the first extensive analysis of the outcomes of cancer biomarker use. Interestingly, they found statistical evidence that biomarker usage has a substantial clinical benefit in cancer patients, even when analyzing biomarkers not yet approved by regulatory authorities. However, Ou et al. [167] “urge oncologists to resist the temptation of adopting unvalidated biomarker findings into practice”. Similarly, Dr. Hayes [178] expressed his concern about biomarker assays not approved by regulatory agencies but extensively used, assuming accuracy and reliability. He often says, “a bad tumor biomarker test is as bad as a bad drug”. Other authors propose what is called an adaptive assessment approach for cancer screening biomarkers based on new high-throughput technologies [137]. In this case, after condensed randomized controlled trials with surrogate endpoints, there would be conditional approval by regulatory authorities and patient access to the screening intervention. Then trials would continue with the definitive endpoint generating more evidence, which would lead to final approval or disapproval. The objection to this approach is that patients would be exposed to the risks associated with premature biomarker test application [137].

In summary, a good cancer biomarker should fulfill all the previously mentioned requirements (Figure 2). Moreover, an ideal biomarker assay should be rapid, preferably binary, easily measurable in an accessible biological specimen, easily adaptable to routine clinical practice, and with a short processing time [167]. Unfortunately, up to now, very few cancer biomarkers, out of all promising biomarkers that have been discovered, have satisfied these rigorous characteristics and therefore have been approved by regulatory agencies. Major impediments in the confirmation of claimed discovered biomarkers and translation to the clinic are, among others, (1) the lack of standardization methods in sample collection, handling, and storage; and (2) the lack of large sample sizes for validation trials, causing a lack of statistical power [179]. These aspects could be overcome by collaborative approaches with multidisciplinary teams involving industry and science, with experts in clinics, biology, epidemiology, statistics, regulation, and healthcare economics [167,179].

## 7. Conclusions and Future Perspectives

Cancer cells undergo multiple changes, and these alterations have been used for decades as cancer biomarkers, mainly tested in tumor tissue. Recent research in the cancer biomarker field has helped in the development of new DNA, RNA, and protein-based cancer biomarkers that can be detected from easily available body fluids. NGS has opened up possibilities for analyzing all cancer-associated genetic alterations in a single assay. Moreover, increased benefits of including analysis of both germline and somatic mutations in a single panel are being recognized for precision oncology. However, most NGS-based tests are optimized for panel, sequencing platform, and site. In addition, although high-throughput transcriptomic and proteomic studies have identified new candidate cancer biomarkers, only very few have been clinically implemented. The biggest challenge in cancer biomarker detection from body fluids is their very low concentration. To overcome this, highly sensitive detection technologies are being developed. For example, nanoparticles with a high surface-to-volume ratio make it possible to attach different molecules to their surface; this, combined with sensor technology, for signal amplification and detection, offers opportunities for the development of more sensitive cancer biomarkers from the body fluids. Finally, it is important to consider all the steps needed to develop a new biomarker. Before clinical implementation, requirements related to analytical and clinical validation, as well as clinical utility, must be fulfilled in order to obtain the necessary regulatory approval by authorities.

## Figures and Tables

**Table 1:** Circulating extracellular-vesicles-associated miRNAs as potential cancer biomarkers.

miRNA	Sample	Cancer	Application	Reference
**Predictive markers of drug response**				
***miR-494-3p***	Plasma EVs	NSCLC	Resistance to osimertinib	Kaźmierczak et al., 2022 [30]
***miR-323-3p*, *miR-1468-3p*, *miR-5189-5p* and *miR-6513-5p***	Plasma EVs	NSCLC	Resistance to osimertinib	Janpipatkul et al., 2021 [31]
***miR-184*, *miR-3913-5p***	Serum EVs	NSCLC	Resistance to osimertinib	Li et al., 2021 [32]
***miR-21***	Plasma ctRNA	*EGFR* mutated NSCLC	Response to EGFR_TKIs	Leonetti et al., 2021 [33]
***miR-125b-5p***	Serum EVs	NSCLC	Predictive of chemotherapy response	Zhang et al., 2020 [34]
***miR-620***	Serum EVs	NSCLC	Predictive of chemotherapy response/Diagnosis	Tang et al., 2020 [35]
***miR-30b*, *miR-328*, and *miR-423***	Plasma EVs	Breast Cancer	Predictive of chemotherapy response	Todorova et al., 2022 [36]
***miR-30a***	Serum EVs	Oral Cancer	Diagnostic, prognostic, cisplatin-resistance	Kulkarni et al., 2020 [37]
**Predictive markers of response to immunotherapy**				
***miR-625-5p***	Plasma EVs	NSCLC	Overall survival after immune checkpoint inhibitor treatment	Pantano et al.2022 [38]
***miR-4649-3p*, *miR-615-3p*, and *miR-1234-3p***	Plasma EVs	Melanoma	Response/prognosis after immune checkpoint inhibitor treatment	Bustos et al., 2020 [39]
**Predictive markers of response to radiotherapy**				
***miR-92a-3p***	Plasma EVs	NSCLC	Resistance to radiotherapy (upregulation)	Zeng et al., 2022 [40]
***miR-96***	Plasma EVs	NSCLC	Radioresistant	Zheng et al., 2021 [41]
**Diagnostic/prognostic markers**				
* (*Let-7b-5p* and *miR-22-3p* and *miR-184*)	Plasma EVs	NSCLC	Diagnostic	Vadla et al., 2022 [42]
***miR-125b-5p* and *miR-5684***	Serum EVs	NSCLC	Diagnostic	Zhang et al., 2020 [34]
***miR-378***	Serum EVs	NSCLC	Prognostic/monitoring	Zhang et al., 2020 [43]
***miR-382***	Serum EVs	NSCLC	Prognostic	Luo et al., 2021 [44]
***miR-1260b***	Plasma EVs	NSCLC	Prognostic	Kim et al., 2021 [45]
***miR-486-5p* and *miR-146a-5p***	Serum EVs	NSCLC	Diagnostic	Wu et al., 2020 [28]
***miR-1246***	Serum EVs	NSCLC	Diagnostic and prognostic	Huang et al., 2020 [46]
***miR-1246 and miR-96***	Plasma EVs	NSCLC	Diagnostic	Zheng et al., 2021 [41]
* (*miR-206*, *miR-24*, *miR-1246,* and *miR-373*)	Plasma EVs	Breast cancer	Diagnostic, combination 98% accuracy	Jang et al., 2021 [47]
***miR-148a***	Serum EVs	Breast cancer	Prognosis	Li et al., 2020 [48]
***miR-138-5p***	Serum EVs	Breast cancer	Prognosis	Xun et al. (2021) [49]
***miR-363-5p***	Plasma EVs	Breast cancer	Prognostic	Wang X et al., 2021 [50]
***miR-1910-3p***	Serum EVs	Breast cancer	Diagnostic (with CA153)	Wang B et al. (2020) [51]
***miR-17-5p***	Serum EVs	Breast cancer	Prognostic	Sueta et al., 2017 [52]
***miR-423*, *miR-424*, *let7-i,* and *miR-660***	Urine EVs	Breast cancer	Diagnostic	Hirschfeld et al., 2020 [53]
***miR-21-5p***	Plasma EVs	Breast cancer	Diagnostic	Liu et al., 2021 [54]
***miR-3662*, *miR-146a*, and *miR-1290***	Serum EVs	Breast cancer	Diagnostic	Li et al., 2021 [55]
***miR-423-3p***	Plasma EVs	Prostate cancer	Predictive of castration-resistance	Guo et al., 2021 [56]
***miR-532-5p***	Urine EVs	Prostate cancer	Prognostic	Kim et al., 2021 [57]
***miR-425-5p***	Plasma EVs	Prostate cancer	Diagnostic of metastatic prostate cancer	Rode et al., 2021 [58]
***miR-16-5p*, *miR-451a*, *miR-142-3p*, *miR-21-5p,* and *miR-636***	Urine EVs	Prostate cancer	Prognostic	Shin et al., 2021 [59]
***miR-125a-5p* and *miR-141-5p***	Plasma EVs	Prostate cancer	Diagnostic	Li et al., 2020 [60]
***miR-375* and *miR-451a***	Urine EVs	Prostate cancer	Diagnostic	Li et al., 2021 [61]
***iR-24-3p***	Saliva EVs	Oral cancer	Diagnostic	He et al., 2020 [62]
***miR-130a***	Plasma EVs	Oral cancer	Diagnostic and prognostic	He et al., 2021 [63]
***miR-126*, *miR-155*, and *miR-21***	Serum EVs	Oral cancer	Diagnostic and prognostic	Chen et al., 2021 [64]
***Let7*, *miR-16,* and *miR-23***	Serum EVs	CRC	Diagnostic	Dohmen et al., 2022 [27]
***miR-139-3p***	Plasma EVs	CRC	Diagnosis	Li et al., 2020 [65]
***miR-126*, *miR-1290*, *miR-23a*, and *miR-940***	Serum EVs	CRC	Diagnostic	Shi et al., 2021 [66]
***miR-4323*, *miR-4284*, *miR-1290*, and *miR-1246***	Serum EVs	CRC	Diagnostic	Handa et al., 2021 [67]
***miR-106b-3p***	Serum EVs	CRC	Diagnostic and prognostic	Liu et al., 2020 [68]
***miR-874***	Serum EVs	CRC	Diagnostic and prognostic	Zhang et al. (2020) [69]
***let-7g* and *miR-193a***	Plasma EVs	CRC	Diagnostic and prognostic	Cho et al. (2021) [70]
***miR-122***	Serum EVs	CRC	Diagnostic of metastatic CRC (liver)	Sun et al., 2020 [26]
***miR-375***	Plasma	Esophageal adenocarcinoma	Prognostic, Overall survival	van Zweeden et al.2021 [71]
***miR-17-5p*** **, *miR-25-3p*, *miR-27a*, *miR-27b*, *miR-191*, *miR-199a-5p*, *miR-211*, *miR-300*, *miR-542-3p*, *miR-586*, *miR-663a***	Plasma/serum	Osteosarcoma	Diagnostic (Higher expression of individual miRNAs)	Meta-analysis by Gao et al., 2020 [72]
***miR-34a*** **, *miR-101*, *miR-124*, *miR-125b*, *miR-139-5p*, *miR-144*, *miR-148a*, *miR-152*, *miR-194*, *miR-195*, *miR-222*, *miR-223*, *miR-326*, *miR-375*, *miR-491-5p***	Plasma/serum	Osteosarcoma	Diagnostic (Lower expression of individual miRNAs)	Meta-analysis by Gao et al., 2020 [72]
* (*miR-21*, *miR-199a-3p*, *miR-143*)	Plasma/serum	Osteosarcoma	Diagnostic (Higher expression, miRNA group)	Meta-analysis by Gao et al., 2020 [72]
* (*miR-199b-5p/miR-124*); (*miR-195-5p*, *miR-199a-3p*, *miR-320a*, *miR-374a-5p*); (*miR-586*, *miR-223*)	Plasma/serum	Osteosarcoma	Diagnostic (Lower expression, miRNA group)	Meta-analysis by Gao et al., 2020 [72]
***miR-21***	Plasma	Diffuse large B-cell lymphoma	Diagnostic (upregulation)	Meta-analysis Lopez-Santillan et al., 2018 [73]
***miR-92a* and *miR-638***	Plasma	Acute lymphoblastic leukemia	Diagnostic	Fayed et al., 2021 [74]
***miR-23b-3p***	Plasma	Hepatocellular carcinoma	Diagnostic (downregulation)	Manganelli et al., 2021 [75]
**Top differentially expressed miRNAs in plasma EVs of cancer patients**				Compiled from the EVmiRNA database (http://bioinfo.life.hust.edu.cn/EVmiRNA, accessed on 20 June 2022) (Liu et al., 2019) [76]
***miR-17-5p*, *miR-3960*, *miR-4488***	Plasma Exo	Breast adenocarcinoma	Diagnostic	
***miR-3168*, *miR-3178*, *miR-425-3p***	Plasma EVs	Breast adenocarcinoma	Diagnostic	
***miR-10a-3p*, *miR-10a-5p*, *miR-1290*, *miR-141-3p*, *miR-183-5p*, *miR-191-5p*, *miR-192-5p*, *miR-194-5p*, *miR-182-5p*, *miR-19b-3p*, *miR-200a-5p*, *miR-200b-3p*, *miR-215-5p*, *miR-19a-3p*, *miR-429***	Plasma Exo	CRC	Diagnostic	
***miR-1224-5p*, *miR-451a***	Plasma EVs	CRC	Diagnostic	
***let-7f-5p*, *let-7g-5p*, *miR-106b-3p*, *miR-1246*, *miR-1260b*, *miR-1290*, *miR-146a-5p*, *miR-155-5p*, *miR-16-2-3p*, *miR-17-5p*, *miR-181a-2-3p*, *miR-20a-5p*, *miR-30e-3p*, *miR-339-5p*, *miR-4488.***	Plasma Exo	Chronic lymphocytic leukemia	Diagnostic	
***miR-126-5p*** **, *miR-182-5p*, *miR-183-5p***	Plasma EVs	Chronic lymphocytic leukemia	Diagnostic	
***miR-103a-3p*** **, *miR-106b-3p*, *miR-10b-5p*, *miR-1307-5p*, *miR-130b-3p*, *miR-142-5p*, *miR-181a-3p*, *miR-186-5p*, *miR-191-5p*, *miR-25-3p*, *miR-423-3p*, *miR-4767*, *miR-877-5p*, *miR-92a-3p*, *miR-92b-3p***	Plasma Exo	Lymphoma	Diagnostic	
***miR-151a-5p*** **, *miR-3195*, *miR-3960*, *miR-4792*, *miR-7641*, *miR-7704.***	Plasma Exo	Oral cancer	Diagnostic	
***miR-1224-5p*, *miR-9-5p***	Plasma Exo	Prostate cancer	Diagnostic	
***miR-1224-5p*, *miR-9-5p***	Plasma EVs	Prostate cancer	Diagnostic	

Exo, exosomes; EVs, extracellular vesicles, including exosomes; NSCLC, non-small cell lung cancer; CRC, colorectal cancer; * combination of miRNAs.

**Table 2:** Commonly studied cancer biomarkers from different sample types. (source: National Cancer Institute [91], and references [16,17].

Biomarker	Cancer	Application	Tumor Tissue/Bone Marrow	Blood	Urine	Stool	Cerebrospinal Fluid	Saliva/Buccal Swab
*ALK* gene rearrangements and overexpression	NSCLC, anaplastic large cell lymphoma, and histiocytosis	To help determine treatment and prognosis	X					
Alpha-fetoprotein (AFP)	Liver cancer and germ cell tumors	To help diagnose liver cancer and follow response to treatment; to assess stage, prognosis, and response to treatment of germ cell tumors		X				
B-cell immunoglobulin gene rearrangement	B-cell lymphoma	To help in diagnosis, to evaluate effectiveness of treatment, and to check for recurrence	X	X				
*BCL2* gene rearrangement	Lymphomas and leukemias	For diagnosis and planning therapy	X	X				
BCR–ABL fusion gene	Chronic myeloid leukemia, acute lymphoblastic leukemia, and acute myelogenous leukemia	To confirm diagnosis, predict response to targeted therapy, help determine treatment, and monitor disease status	X	X				
Beta-2-microglobulin (B2M)	Multiple myeloma, chronic lymphocytic leukemia, and some lymphomas	To determine prognosis and follow response to treatment		X	X		X	
Beta-human chorionic gonadotropin (Beta-hCG)	Choriocarcinoma and germ cell tumors	To assess stage, prognosis, and response to treatment		X	X			
Bladder Tumor Antigen (BTA)	Bladder cancer and cancer of the kidney or ureter	As surveillance with cytology and cystoscopy of patients already known to have bladder cancer			X			
BRAF gene V600 mutations	Cutaneous melanoma, Erdheim–Chester disease, Langerhans cell histiocytosis, CRC, and NSCLC	To help determine treatment	X					
*BRCA1* and *BRCA2* gene mutations	Ovarian and breast cancers	To help determine treatment	X	X				
CA15-3/CA27.29	Breast cancer	To assess whether treatment is working or if cancer has recurred		X				
CA19-9	Pancreatic, gallbladder, bile duct, and gastric cancers	To assess whether treatment is working		X				
CA-125	Ovarian cancer	To help in diagnosis, assessment of response to treatment, and evaluation of recurrence		X				
CA27.29	Breast cancer	To detect metastasis or recurrence		X				
Calcitonin	Medullary thyroid cancer	To help in diagnosis, check whether treatment is working, and assess recurrence		X				
Carcinoembryonic antigen (CEA)	CRC and some other cancers	To monitor the effectiveness of treatment and to detect recurrence or spread		X				
CD19	B-cell lymphomas and leukemias	To help in diagnosis and to help determine treatment	X	X				
CD20	Non-Hodgkin lymphoma	To help determine treatment		X				
CD22	B-cell lymphomas and leukemias	To help in diagnosis and to help determine treatment	X	X				
CD25	Non-Hodgkin (T-cell) lymphoma	To help determine treatment		X				
CD30	Classic Hodgkin lymphoma, and B-cell and T-cell lymphomas	To help determine treatment	X					
CD33	Acute myeloid leukemia	To help determine treatment		X				
Chromogranin A (CgA)	Neuroendocrine tumors	To help in diagnosis, assessment of treatment response, and evaluation of recurrence		X				
Chromosome 17p deletion	Chronic lymphocytic leukemia	To help determine treatment		X				
Chromosomes 3, 7, 17, and 9p21	Bladder cancer	To help in monitoring for tumor recurrence			X			
Circulating tumor cells of epithelial origin (CELLSEARCH)	Metastatic breast, prostate, and CRC	To inform clinical decision-making, and to assess prognosis		X				
C-kit/CD117	Gastrointestinal stromal tumor, mucosal melanoma, acute myeloid leukemia, and mast cell disease	To help in diagnosis and to help determine treatment	X	X				
Cyclin D1 (*CCND1*) gene rearrangement or expression	Lymphoma and myeloma	To help in diagnosis	X					
Cytokeratin fragment 21-1	Lung cancer	To help in monitoring for recurrence		X				
Des-gamma-carboxy prothrombin (DCP)	Hepatocellular carcinoma	To monitor the effectiveness of treatment and to detect recurrence		X				
*DPD* gene mutation	Breast, CRC, gastric, and pancreatic cancers	To predict the risk of a toxic reaction to 5-fluorouracil therapy		X				
*EGFR* gene mutation	NSCLC	To help determine treatment and prognosis	X					
Estrogen receptor (ER)/progesterone receptor (PR)	Breast cancer	To help determine treatment	X					
*FGFR2* and *FGFR3* gene mutations	Bladder cancer	To help determine treatment	X					
Fibrin/fibrinogen	Bladder cancer	To monitor progression and response to treatment			X			
*FLT3* gene mutations	Acute myeloid leukemia	To help determine treatment		X				
FoundationOne CDx (F1CDx) genomic test	Any solid tumor	As a companion diagnostic test to determine treatment	X	X				
Gastrin	Gastrin-producing tumor (gastrinoma)	To help in diagnosis, monitor the effectiveness of treatment, and detect recurrence		X				
Guardant360 CDx genomic test	Any solid tumor	As a companion diagnostic test to determine treatment and for general tumor mutation profiling		X				
HE4	Ovarian cancer	To plan cancer treatment, assess disease progression, and monitor for recurrence		X				
*HER2/neu* gene amplification or protein overexpression	Breast, ovarian, bladder, pancreatic, and stomach cancers	To help determine treatment	X					
5-HIAA	Carcinoid tumors	To help in diagnosis and to monitor disease			X			
*IDH1* and *IDH2* gene mutations	Acute myeloid leukemia	To help determine treatment	X	X				
Immunoglobulins	Multiple myeloma and Waldenström macroglobulinemia	To help diagnose disease, assess response to treatment, and look for recurrence		X	X			
*IRF4* gene rearrangement	Lymphoma	To help in diagnosis	X					
*JAK2* gene mutation	Certain types of leukemia	To help in diagnosis	X	X				
*KRAS* gene mutation	CRC and NSCLC	To help determine treatment	X					
Lactate dehydrogenase	Germ cell tumors, lymphoma, leukemia, melanoma, and neuroblastoma	To assess stage, prognosis, and response to treatment		X				
Mammaprint test (70-gene signature)	Breast cancer	To evaluate risk of recurrence	X					
Microsatellite instability (MSI) and/or deficient mismatch repair (dMMR)	CRC and other solid tumors	To guide treatment and to identify those at high risk of certain cancer-predisposing syndromes	X					
*MYC* gene expression	Lymphomas and leukemias	To help in diagnosis and to help determine treatment	X					
*MYD88* gene mutation	Lymphoma and Waldenström macroglobulinemia	To help in diagnosis and to help determine treatment	X					
Myeloperoxidase (MPO)	Leukemia	To help in diagnosis		X				
Neuron-specific enolase (NSE)	Small cell lung cancer and neuroblastoma	To help in diagnosis and to assess response to treatment		X				
*NTRK* gene fusion	Any solid tumor	To help determine treatment	X					
Nuclear matrix protein 22	Bladder cancer	To monitor response to treatment			X			
Oncotype DX Breast Recurrence Score test (21-gene signature)	Breast cancer	To evaluate risk of distant recurrence and to help plan treatment	X					
Oncotype DX Genomic Prostate Score test (17-gene signature)	Prostate cancer	To predict the aggressiveness of prostate cancer and to help manage treatment	X					
OVA1 test (5-protein signature)	Ovarian cancer	To pre-operatively assess pelvic mass for suspected ovarian cancer		X				
PCA3 mRNA	Prostate cancer	To determine need for repeating biopsy after a negative biopsy			X			
PML/RARα fusion gene	Acute promyelocytic leukemia	To diagnose, to predict response to all-trans-retinoic acid or arsenic trioxide therapy, to assess effectiveness of therapy, monitor minimal residual disease, and predict early relapse	X	X				
Programmed death ligand 1 (PD-L1)	NSCLC, liver cancer, stomach cancer, gastroesophageal junction cancer, classical Hodgkin lymphoma, and other aggressive lymphoma subtypes	To help determine treatment	X					
Prolaris test (46-gene signature)	Prostate cancer	To predict the aggressiveness of prostate cancer and to help manage treatment		X				
Prostate-specific antigen (PSA)	Prostate cancer	To help in diagnosis, to assess response to treatment, and to look for recurrence		X				
Prostatic Acid Phosphatase (PAP)	Metastatic prostate cancer	To help in diagnosing poorly differentiated carcinomas		X				
*ROS1* gene rearrangement	NSCLC	To help determine treatment	X					
Soluble mesothelin-related peptides (SMRP)	Mesothelioma	To monitor progression or recurrence		X				
Somatostatin receptor	Neuroendocrine tumors affecting the pancreas or gastrointestinal tract	To help determine treatment	X					
T-cell receptor gene rearrangement	T-cell lymphoma	To help in diagnosis; sometimes to detect and evaluate residual disease	X	X				
Terminal transferase (TdT)	Leukemia and lymphoma	To help in diagnosis	X	X				
Thiopurine S-methyltransferase (TPMT) enzyme activity or *TPMT* genetic test	Acute lymphoblastic leukemia	To predict the risk of severe bone marrow toxicity (myelosuppression) with thiopurine treatment		X				X
Thyroglobulin	Thyroid cancer	To evaluate response to treatment and to look for recurrence		x				
UGT1A1*28 variant homozygosity	CRC	To predict toxicity from irinotecan therapy		X				X
Urine catecholamines: VMA and HVA	Neuroblastoma	To help in diagnosis			X			
Urokinase plasminogen activator (uPA) and plasminogen activator inhibitor (PAI-1)	Breast cancer	To determine the aggressiveness of cancer and guide treatment	X					
DNA methylation markers based on References [16,17]								
Methylation of *MGMT* promoter	Glioblastoma	Drug response to chemotherapy	X					
Methylation of *MLH1*	Lynch syndrome	Treatment decision	X					
Methylation of *NDRG4* and *BMP3*	Colorectal Cancer	Diagnostic				X		
Methylation of *SEPT9* promoter	Colorectal Cancer	Diagnostic			X			

NSCLC, non-small cell lung cancer; CRC, colorectal cancer; UGT1A1*28, variant with seven (TA) repeats; X, detected in sample.

**Table 3:** List of technologies used for biomarker discovery and detection, and their applications, advantages, and disadvantages.

Technology	Applications	Advantages	Disadvantages
FISH	Detection of chromosomal abnormalities	Cell-based genetic results, specificity, simplicity, and reliability	Unable to detect sequence mutations
PCR/real-time PCR/digital PCR	Detection of targeted sequence mutations, gene fusions, or DNA methylation	High sensitivity and specificity, simplicity, good reproducibility, suitable in a clinical setting, and low cost	Restricted to targeted mutations and limited throughput
NGS	Detection of somatic and germline alterations in a large number of genes	High-throughput tool; can be targeted or genome-wide, and can detect different types of genetic alterations at the same time	Results depend on the platform. Difficult to interpret the significance of low-frequency variants. Genome-wide approaches require bioinformatic analysis. Site-specific testing for clinical applications
Flow cytometry	Cell count and identification, DNA quantification	High sensitivity and rapid analysis	Restricted to specific parameters
Gene expression microarrays	Differences in gene expression between tumor subtypes or between tumor and normal tissue or in tumor tissue before and after treatment, etc.	High-throughput tool	Bioinformatic analysis is required. Not all targets are identified
IHC	Detection of protein expression	Localization of protein expression in the tumor tissue	Restricted to proteins with available antibodies. Subjective interpretation
ELISA	Detection of protein expression, primarily in body fluids	Easy procedure and quantitative	Restricted to proteins with available antibodies. Limited detection sensitivity in body fluids
Lectin microarrays	Glycomic profiling	Can be useful in tumor tissues and body fluids, high-throughput tool, high sensitivity, and rapid analysis	Inconsistencies due to variation between batches and between purification procedures
MS	Protein profiling of tumor tissues or body fluids	Can be used for targeted assays or biomarker discovery, and highly multiplex	Procedure complexity, low sensitivity, and throughput
RPPA	Targeted detection of protein levels	High reproducibility, high throughput, and lower cost than MS	Need for special devices, restricted to proteins with validated antibodies
Biosensors/nanotechnology	Detection of low concentration biomarkers primarily in body fluids	High sensitivity and specificity, and ease of use	Low stability, poor reproducibility, problems in miniaturizing devices, and low performance in human whole blood samples
Microfluidics	Detection of low concentration biomarkers primarily in body fluids	High sensitivity, high throughput, cost-effective tool, and can be combined with biosensors	Needs improvement in accuracy and efficiency
CRISPR-based ctDNA/RNA detection	Detection of low concentration biomarkers primarily in body fluids	High sensitivity and specificity; can be combined with biosensors	Complicated multi-step procedure; lack of high-throughput design
Synthetic biomarker technology	Sensing of dysregulated activity of tumor cells or tumor microenvironment	Molecular amplification of tumor biomarker	Significant noise from off-target activity; need for better knowledge on early stage cancer pathogenesis

FISH, fluorescence in situ hybridization; PCR, polymerase chain reaction; NGS, next-generation sequencing; IHC, immunohistochemistry; ELISA, Enzyme-Linked ImmunoSorbent Assay; MS, mass spectrometry; RPPA, reverse-phase protein arrays; CRISPR, clustered regularly interspaced short palindromic repeats; ctDNA/RNA, circulating tumor DNA/RNA.

**Table 4:** Definitions of terms assessed in analytical validation, according to References [171,172].

Term	Definition
Analytical Sensitivity	The smallest concentration of a substance in a biological specimen that can be reliably measured by an analytical procedure
Analytical Specificity	The ability of an assay to measure the specific substance (intended target), rather than others, in a biological specimen
Analytical Accuracy	The closeness of agreement between the value which is accepted either as a conventional true value or an accepted reference value and the value found. Usually, there is a comparison with another measurement technique
Analytical Repeatability	A measure of the extent to which a test conducted multiple times on the same subject, in the same laboratory, using the same equipment, by the same operator, over a short period of time, gives the same result
Analytical Reproducibility	A measure of the extent to which a test conducted multiple times in different laboratories, using different equipment, by different operators, or over different periods of time, gives comparable results

**Table 5:** Definitions of terms assessed in clinical validation, according to References [167,171,172,175].

Term	Definition
Diagnostic Sensitivity	The measure of how often a binary biomarker test correctly indicates the presence of a particular characteristic in individuals that truly have the characteristic. Biomarker sensitivity is the number of true positive results divided by the number of true-positive plus false-negative results.
Diagnostic Specificity	The measure of how often a binary biomarker test correctly indicates the absence of a particular characteristic in individuals who truly do not have the characteristic. Biomarker specificity is the number of true-negative results divided by the number of true-negative plus false-positive results.
Positive predictive value	The measure of how often a binary biomarker test correctly indicates the presence of a particular characteristic in individuals that have a positive test result. Biomarker positive predictive value is the number of true positive results divided by the number of true-positive plus false-positive results.
Negative predictive value	The measure of how often a binary biomarker test correctly indicates the absence of a particular characteristic in individuals that have a negative test result. Biomarker negative predictive value is the number of true negative results divided by the number of true-negative plus false-negative results.
Receiver operating characteristics (ROC) curve	Plot showing the relationship between sensitivity (true positive) and 1-specificity (true negative). It is a graphical way of describing likelihood ratios at various values of the biomarker test.
Area under the ROC curve (AUC^ROC^)	The ability of a binary biomarker to distinguish two or more groups of individuals. It is a measure of discrimination. Values range from 0 to 1, and 1 corresponds to perfect discriminative power.

## Data Availability

Not applicable.
